# Fabrication of a Large-Area, Fused Polymer Micromold Based on Electric-Field-Driven (EFD) μ-3D Printing

**DOI:** 10.3390/polym11111902

**Published:** 2019-11-18

**Authors:** Zilong Peng, Nairui Gou, Zilong Wei, Jiawei Zhao, Fei Wang, Jianjun Yang, Yinan Li, Hongbo Lan

**Affiliations:** Shandong Engineering Research Center for Additive Manufacturing, Qingdao University of Technology, Qingdao 266520, China

**Keywords:** additive manufacturing, EFD μ-3D printing, polymers, large-area micromolds

## Abstract

An electric-field-driven (EFD), μ-3D printed, fused polymer technique has been developed for the fabrication of large-area microscale prototype molds using typical polymer materials, including microcrystalline wax (MC-wax), polycaprolactone (PCL), and polymathic methacrylate (PMMA). This work proposes an alternative for large area microscale modes and overcomes the limitation of high cost in the traditional mold manufacturing industry. The EFD principle enables printing of fused polymers materials more than one order of magnitude lower than the nozzle diameter, contributing to the necking effect of the Taylor cone jet, which is the key factor to achieve the microscale manufacturing. Numerical simulation of electric field distribution between the meniscus and substrate was carried out to elucidate the dependence of electric field distribution on the meniscus condition of three types of polymers under printable voltage, and the electrical field parameters for the EFD μ-3D printing were determined. A number of experiments were printed successfully using a large range of viscosity materials, ranging from tens of mPa·s to hundreds of thousands of mPa·s of PCL and PMMA. The differences in parameters of different materials, such as viscosity, tensile properties, and surface energy, were studied to assess their use in different fields. Using proper process parameters and a nozzle with an inner diameter of 200 μm, three different application cases were completed, including a Wax microarray and microchannel with a minimum dot diameter of 20 μm, a PCL mesh structure with a minimum line width of 5 μm, and a PMMA large-area mold with a maximum aspect ratio of 0.8. Results show that the EFD μ-3D printing has the outstanding advantages of high printing resolution and polymer material universality.

## 1. Introduction

Micro- and nanoscale additive manufacturing is a research hotspot and it experiences technical difficulty in the current manufacturing industry. It has been widely applied in biomedical fields, tissue engineering [1], new materials (composite materials, functionally graded materials, etc.) [2], micro- and nano-optical devices, sensors [3,4], microfluidic devices, and printing electronics [5,6,7].

The main manufacturing methods for microscale molds include hot embossing [8,9], injection molding [10,11,12], laser ablation [13,14], and LIGA (Lithographie, Galvanoformun and Abformung) [15]. However, these technologies basically rely on high-precision prototype molds, which are costly when obtaining satisfactory manufacturing accuracy, especially for microscale molds [16,17]. In order to reduce the manufacturing cost of prototype mold preparation, the process of fabricating a convex mold using the micro- and nanoscale additive manufacturing technology is studied.

Fused polymers are some of the most widely used 3D printing materials [18,19]. Materials such as polymathic methacrylate (PMMA) have good aspect ratios and can be used to make micro- and nanoscale molds [20]. Polycaprolactone (PCL) has good biodegradability and has the potential to produce biological scaffolds [21]. As for MC-Wax, it can obtain good surface quality under reasonable solidification control, and can solve the problem cost-effective manufacturing of large-scale molds [22].

At present, technology for direct 3D printing of polymers for fabricating convex molds mainly includes fused deposition modeling (FDM) [23,24], multi-jet fusion (MJF) [25], electrospinning [26,27], and electrohydrodynamic jet (E-jet) printing [28,29]. FDM is the most widely used additive manufacturing technology, with the advantages of having a wide range of printing materials, simple operation, and low cost. However, it is hard to reach a printing resolution lower than 100 μm, as the extrusion material is limited [30,31]. MJF can produce high-resolution structures by using multiple nozzles, giving high efficiency and a large printing size. Electrospinning is based on an unstable jet whip effect, making it difficult to control the processing for complex structures [32]. E-jet printing is a new material jet printing technology that uses an electric field to “pull” a very fine jet from the tip of the nozzle. Through applying a high voltage between the conductive nozzle and conductive substrate, a strong electric force that forms between the two electrodes pulls the liquid out of the nozzle to form a Taylor cone. E-jet printing can obtain high print resolution, which eliminates the nozzle diameter limitations for printing accuracy. However, the materials used for E-jet printing mainly depend on solution polymerization, the preparation of materials does not have uniform criteria, and the printing process parameters are usually based on the given materials. Moreover, E-jet printing still has problems, such as complex preparation of printing solutions, limitation of substrate conductivity, solvent volatilization residues, and low surface quality caused by solvent volatilization [33,34,35].

In this paper, a large-area micromold produced using EFD μ-3D printing technology for fused polymer materials has been developed, which can be used to improve mold accuracy and reduce the manufacturing cost of traditional high precision molds. Three types of polymers with wide material universality, namely microcrystalline wax (MC-wax), polycaprolactone (PCL), and polymathic methacrylate (PMMA) have been printed for precision prototype mold fabrication. The printing process features for the three type of fused polymer materials have been researched. Then, three different application cases have been developed based on the materials′ printing features. A wax microarray and microchannel with a minimum dot diameter of 20 μm, a PCL mesh structure with a minimum line width of 5 μm, and a PMMA large-area mold with a maximum aspect ratio of 0.8 were obtained successfully. This method is supposed to improve the mold accuracy and reduce the manufacturing costs of traditional high precision molds.

## 2. Materials and Methods

### 2.1. Materials

The printing materials used in this study were MC-Wax, PCL, and PMMA. The viscosity of the molten materials was measured using an Antor Paar MCR302 rheometer (Shanghai, China); the specific parameters are shown in Table 1. Glass microscope slides, Shitai CITOGLAS 10127101P-G (Nantong, China) with a thickness of 2 mm were selected as the printing substrates. An Olympus DSX-510 optical digital microscope (Tokyo, Japan) was applied to observe the sample structure, for which the maximum observation of magnification was 9000×. A high-speed camera, iSpeed-221 (Rochford, Essex, UK) capturing 204,100 frames per second was used to observe the 3D printing process.

In our research, the melting point and the viscosity of the material are two important factors that influence the 3D printing process. In Table 1, we can see that the melting points of MC-Wax and PCL are similar, while that of PMMA is relative higher at 200 °C. The relative molecular mass of MC-Wax ranges from 500 to 1000 and the relative molecular mass of PMMA ranges from 80,000 to 120,000, which mainly reflects the viscosity of polymers in the melting state. The viscous flow of the polymer mainly depends on the displacement of the center of gravity of the molecular chain along the flow direction and the slip between the chains. With the increase of relative molecular mass, the number of segments in a molecular chain will increase, resulting in greater chain synergy to complete the migration of the center of gravity. The shear viscosity of the polymer melt increases with increasing molecular mass. Materials with a large molecular weight have poor fluidity and high apparent viscosity. The larger the molecular mass is, the larger the viscosity will be.

### 2.2. Methods

**Figure 1:**
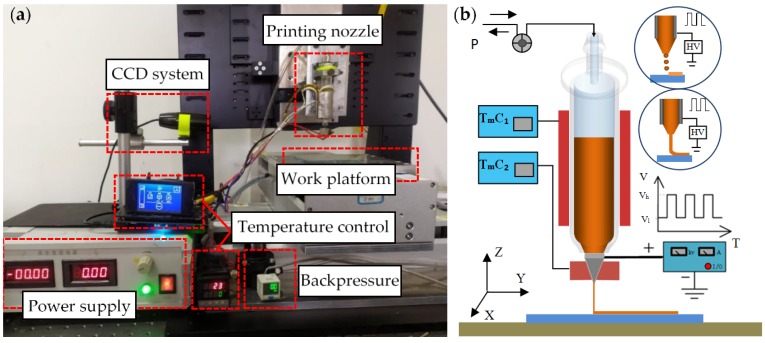
The experimental set up and principle of electric-field-driven (EFD) μ-3D printing: (**a**) the experimental devices. CCD = charge coupled device; (**b**) schematic of the EFD μ-3D printing technology, HV = high voltage.

The principle of EFD μ-3D printing fused polymers is shown in Figure 1. The machining system included a power supply module, a full closed loop dual heating module, a backpressure regulation module, a three axes motion control device, and a charge coupled device (CCD) system for printing observation. The power module could output a 0–3600 V direct current voltage or pulse width adjustable pulse voltage.

The double heating module was composed of a storage barrel and a metal nozzle, and they were heated separately by a thermal resistor. The temperature was controlled by thermo-sensitive feedback. The syringe used was glass, which had good insulation and was easy to replace. A metal nozzle was used with an inner diameter of 250 μm and an outer diameter of 500 μm. The backpressure regulation module adjusted the pressure through a precision pressure pump. The CCD camera was used to observe the printing process in real-time. The motion control device was composed of linear motors in the X axis and Y axis directions and a high precision servo motor in Z axis direction. The repeated positioning accuracy reached up to ±5 μm.

The operation process is as follows. Firstly, after opening the ring heater, the printing material was heated in the storage barrel to a molten state. Secondly, further heating of the molten material through the nozzle heater was performed to control the temperature precisely, which ensured the exact viscosity characteristics of the material. Thirdly, the melted material formed a meniscus at the nozzle tip under the effect of its own gravity, the surface tension, and applied backpressure (including positive and negative pressure). Lastly, the high-voltage power supply was opened and the meniscus was stretched into a cone jet under the action of the electric field, which was finally deposited on the target substrate.

**Figure 2:**
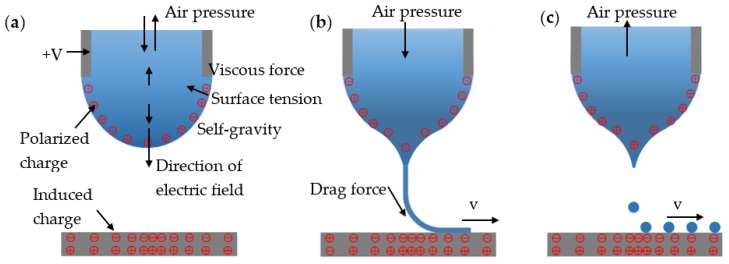
Formation mechanism of the meniscus: (**a**) force analysis of the meniscus and electric field formation; (**b**) high-viscosity material deposition with positive air pressure and drag force form between the cone jet and substrate; (**c**) low-viscosity material with negative air pressure.

Figure 2 shows that the forming principle of the meniscus and the formation mechanism of the electric field. The state of the droplets at the nozzle is mainly influenced by viscous force, surface tension, self-gravity, and back pressure. The backpressure can be divided into positive pressure and negative pressure, according to the material viscosity. For high-viscosity materials such as PCL and PMMA, due to the large viscous force between materials the fluidity is poor, so it is necessary to apply positive pressure to extrude the material to the nozzle. For low-viscosity materials such as MC-Wax, the molten material will form a large drop at the tip of the nozzle freely under the action of its own gravity. To avoid dropping, it is necessary to apply a certain negative pressure to balance its self-gravity.

The conductive nozzle is connected to the positive pole of the high voltage power supply. The droplet at the nozzle is polarized and generates a positive charge under high voltage. The surface charge of the substrate is rearranged due to electrostatic induction between the substrate and the nozzle, the negative charge is distributed on the upper surface, and the positive charge is located on the lower surface. The overall charge distribution shows a state of gradual dispersion from the center to both sides and forms a self-excited electrostatic field between the nozzle and the substrate. This differs from traditional electrohydrodynamic jet printing, which requires an electrode pair; that is, the conductive nozzle and the conductive substrate are connected to the positive and the negative poles of the high voltage power supply, respectively. EFD μ-3D printing only needs the conductive nozzle to connect with the positive power pole, and the grounded substrate is no longer needed as the counter electrode. The stable electric field (self-excited electrostatic field) required to form the cone jet by electrostatic induction is realized. As a single-potential, self-excited EFD μ-3D printing process, this breaks through the requirements and limitations of conventional electrohydrodynamic jet printing for height formation, material type, substrate conductivity, and flatness, especially in large-dimensional components and conformal or curved 3D printing, which have unique advantages in achieving integrated macro- and microscale structure printing.

After applying the voltage, under the action of the electric field force, the polarized droplet with the positive charge is stretched and deformed by the overall downward force to form a Taylor cone. As the voltage further increases, the electric field force drives the droplet, breaking through the surface tension and forming a very fine cone jet. For polymer materials with different viscosity, the deformations of the jet can be divided into continuous cone jets and fracture cone jets. For high viscosity materials, due to the large viscous force, the jet can be stretched to a very fine state without breaking, and is deposited in the form of continuous microfilaments. For low-viscosity materials, the jet will rupture at a certain frequency and aggregate into droplets.

## 3. Results and Discussions

### 3.1. Numerical Simulation

The finite element simulation software , COMSOL Multiphasics (Stockholm, Sweden) was used to analyze the electric field distribution and electric field intensity around the nozzle and substrate when printing different materials, revealing the mechanism of EFD μ-3D printing.

**Figure 3:**
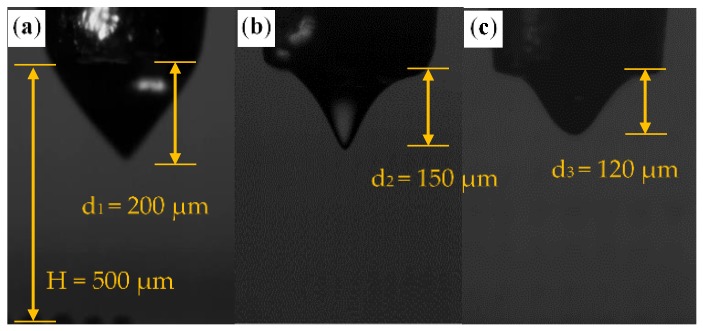
Different sizes of Taylor cones from different materials recorded by a high-speed camera: (**a**) microcrystalline wax (MC-Wax); (**b**) polycaprolactone (PCL); (**c**) polymathic methacrylate (PMMA).

In order to study the electric field distribution of different materials in the simulation model, MC-Wax, PCL, and PMMA are taken as research examples. In the simulation, the relative permittivity of different materials and the size of the Taylor cone are incorporated into the electric field calculation. The elongation length of the three types of Taylor cones recorded by the high-speed camera is shown in Figure 3. Through studying the electric field distribution of different materials in different Taylor cone states, the electric field strength of the cone jet is studied and theoretical guidance is provided for the subsequent experiments.

**Figure 4:**
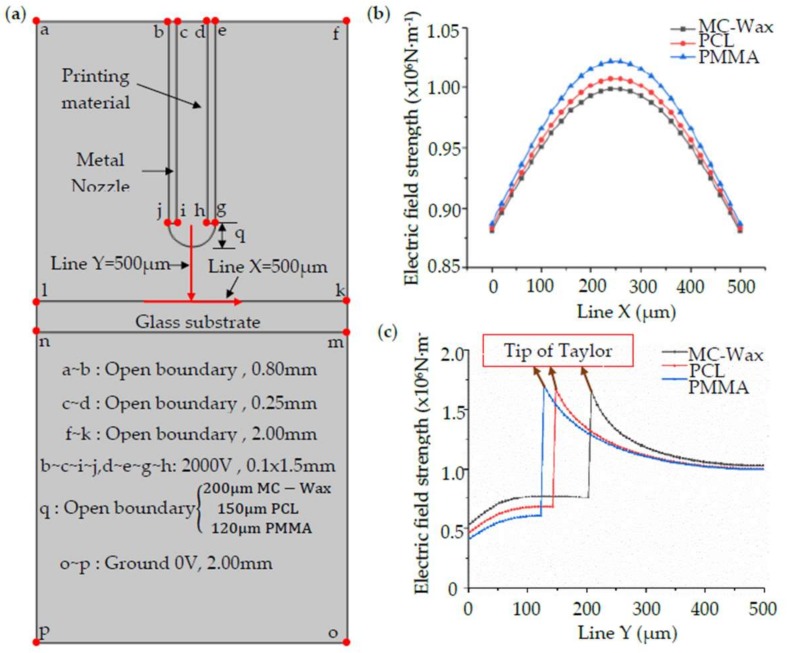
Simulation geometry model and electric field comparison chart: (**a**) simulation geometry model and boundary conditions; (**b**) electric field strength at the X position; (**c**) electric field strength at the Y position.

The printed substrate is composed of insulated glass and the nozzle is made from metal. In the preview simulation of printing materials, a cone jet was generated at a voltage of 2000 V and distance of 500 μm. According to this, the established geometric model is shown in Figure 4a.

The simulation adopts a two-dimensional geometry model. The simulation geometry model and the boundary conditions are shown in Figure 4a. The areas of b–c–i–j and d–e–g–h are related to the metal nozzle, with an inner diameter of 250 μm and an outer diameter of 350 μm, and a voltage of 2000 V is applied to it. The area of k–l–n–m is the glass substrate, with a thickness of 200 μm. The boundary of o–p is grounded, and the voltage is 0 V. The other field in this model is defined as air.

Since the simulation of the electrostatic field does not involve a numerical change in time, the electric field distribution is approximately instantaneous, so the steady state study is selected to solve the static electric field distribution. The model is triangulated, and the degree of subdivision is extremely refined. The maximum cell size of the triangular mesh is set to 0.1 mm, and the minimum cell size is set to 0.1 μm. Finally, a consortium of 6 solid objects is formed. The stereotyped geometry has a complete mesh composed of 2482 domain units and 406 boundary units.

Line X and Y are the reference lines selected to analyze the change of electric field strength in the spacing between the nozzle and the substrate, and the arrow represents the positive direction. For different materials, the electric field distribution and strength along line X and line Y are as shown in Figure 4b,c, respectively.

### 3.2. Electric Field Strength

The voltage value and the distance mainly determine the electric field strength in the experiment. The voltage primarily provides the electric field force required for the droplet stretching, and the voltage for printing is inversely proportional to the nozzle-to-substrate distance. The electric field strength can be roughly estimated by Equation (1):

(1) $$E=\frac{4U}{D_{n}\left(ln\left(8H/D_{n}\right)\right)}$$

where *E* is the electric field strength, *U* is the voltage applied between the metal nozzle and the substrate, *D*_n_ is the diameter of the nozzle, and *H* is the distance between the nozzle and substrate. At a certain distance, the meniscus is stretched to form a Taylor cone as the voltage increases. When the voltage is further increased, the electric field force overcomes the surface tension and stretches into a cone jet at the tip of the Taylor cone, which finally forms a wire or droplet deposit on the target substrate.

**Figure 5:**
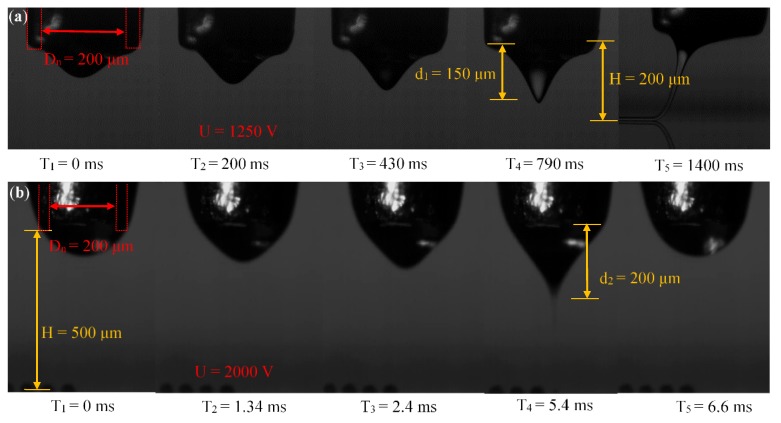
Cone jet deformation and deposition process: (**a**) high-viscosity material (PCL); (**b**) low-viscosity material (MC-Wax).

The printing distance is calculated from the tip of the nozzle to the substrate. The shape of the meniscus and the length of the cone jet are taken into consideration to determine the appropriate distance. When the distance is too small, the meniscus will contact the substrate, which leads to the deformation process of the jet being compressed, and the print quality will be variable. If the voltage is too high it will make the jet deformation more complex in the air and will make the cone jet prone to producing irregular motion, such as jitter during deposition, which affects printing stability. The deformation and deposition processes of the jet are shown in Figure 4. The printing distance generally ranges from 150 to 500 μm, depending on the material viscosity. For high-viscosity materials, as shown in Figure 5a, the cone jet is stretched by the electric field force and the stretched length is *d*_1_ of 150 μm, indicating that the minimum printing distance shall be bigger than *d*_1_. For low-viscosity materials, as shown in Figure 5b, the cone jet of the material undergoes deformation, fracture, and aggregation to form droplets in the air, and the deformation and fracture distance is *d*_2_ of 200 μm. Taking the time for the cracked jet to form a droplet into consideration, the distance is generally selected to be 400–500 μm.

**Figure 6:**
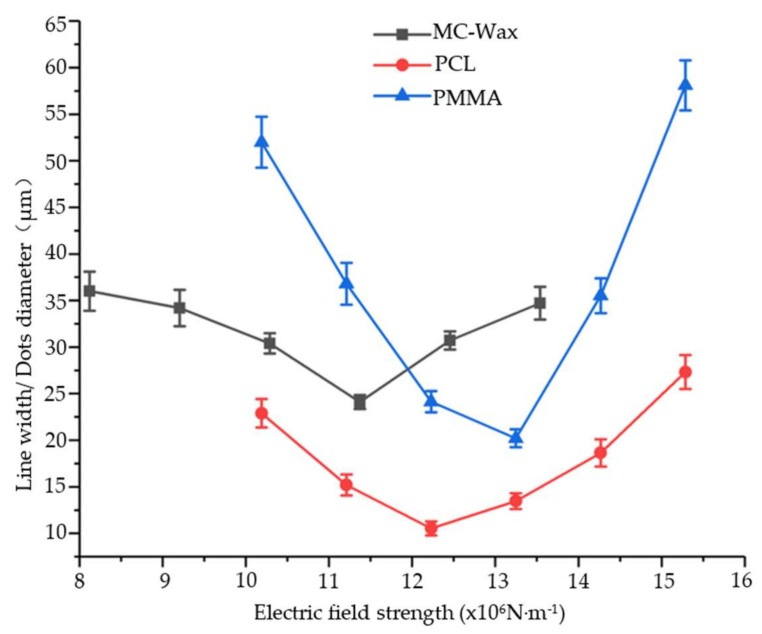
Effect of electric field strength on print line width and diameter.

The electric field strength required for printing totally depends on the interfacial tension of the meniscus; that is, the viscosity of the material is critical. For low-viscosity materials, such as MC-Wax, continuous and stable printing can be achieved at a strength of 8–13.5 × 10^6^ N/m, equal to the voltage of 1500–2000 V at the distance of 500 μm. For high-viscosity materials, such as PCL and PMMA, the electric field strength needs to be increased to 10–15.6 × 10^6^ N/m, equal to the voltage of 1000–1500 V at the distance of 250 μm. When the electric field strength is too low, the cone jet cannot be formed and the printing is discontinuous. Within the appropriate parameters, the cone jet can be freely generated and can ensure stable printing, but the diameter and line width of the printed dots are slightly different, as shown in Figure 6. As the electric field strength increases, the point diameter and line width tend to decrease first and then increase, and the magnitude of the change is slightly different due to the difference in viscous force. The changing amplitude grows as the viscous force increases. While the electric field strength exceeds the printable range, the dislocation of dots and bending of the line will appear during printing. At a lower electric field strength, the electric field force of the micro molten material is too small and is unable to overcome the surface tension. When the electric field strength is within the printable range, a fine cone jet can be formed to achieve stable printing. However, when a certain value is exceeded, the microjet affected by the electric field in the air tends to whip (or swing) in the air and even leads to multiple jets, which highly influences the printing stability and makes it difficult to achieve accurate and controllable 3D printing. Therefore, for the EFD μ-3D printing, the applicable material range is wide and the quality of the printed microfilament can be controlled by adjusting the voltage value.

### 3.3. Print Speed

**Figure 7:**
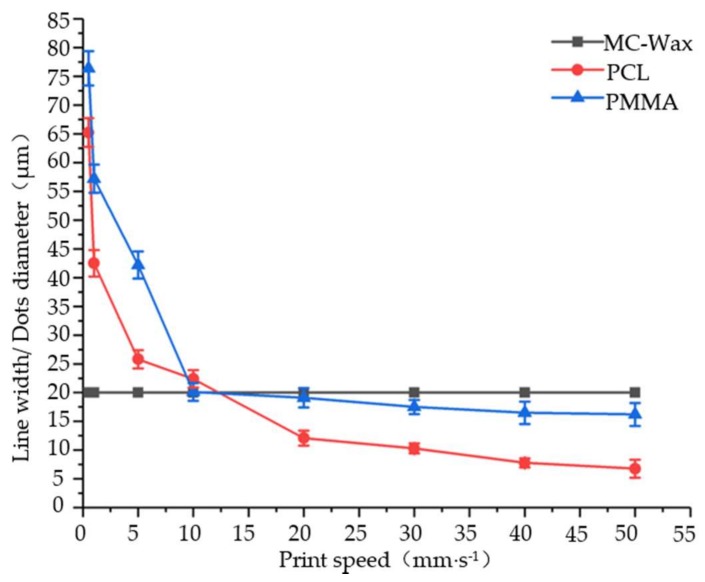
Effect of print speed on line width and diameter of the print dots.

The print speed (the speed of worktable movement) has an important influence on the diameter and line width of the print dots. The effect of print speed as assessed by experimental results is shown in Figure 7. By analyzing the tendency of the curve, it is known that for high-viscosity materials such as PCL and PMMA, as the printing speed increases, the line width decreases accordingly. Since the deposition material is bonded to the substrate, a pulling force (viscous drag force) is generated on the microwires of the deposition gap, which forces the microwires to stretch to further thin the filaments. While printing speed increases within a certain range, the fine filaments are not broken because of their good toughness. When the printing speed is too low, the pulling force caused by the viscous drag force is so low that it has little influence on the line width. However, as the printing speed increases to a high degree and beyond the tensile limit, the continuity of the line will be broken. For low viscosity materials, the printing speed mainly affects the spacing or coincidence rate of the points, but has little effect on the diameter of the points.

### 3.4. Substrate Temperature

**Figure 8:**
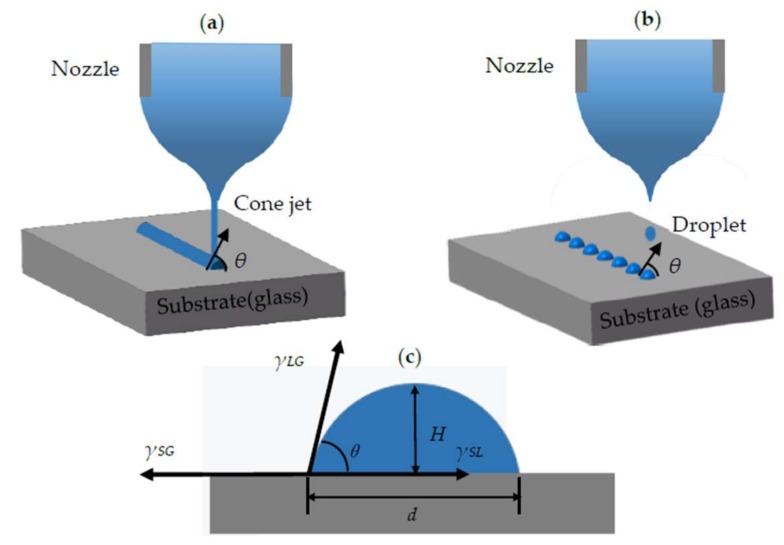
Schematic diagram of droplet deposition: (**a**) high-viscosity material deposition on the glass substrate; (**b**) low-viscosity material deposition on the glass substrate; (**c**) model of the contact angle on the substrate.

As the droplet is in free space, it will remain spherical due to the presence of interfacial tension. However, when the liquid is in contact with the solid plane, its final shape totally depends on the cohesive force inside the liquid and the relative amount of adhesion between the liquid and the solid. The liquid will automatically spread over the solid surface or present at a certain angle of contact when placed on a solid surface. In this experiment, the jets stretched from the Taylor cone are either cylindrical or break to form a spherical droplet due to the interfacial tension, as shown in Figure 8a,b. The angle between the air and the glass surface is *θ*.

It is assumed that different interface forces can be expressed by the tension acting in the interface direction. As the liquid maintains the equilibrium position on the solid plane, the sum of the component forces of the interfacial tension in the horizontal direction should be equal to zero, as in Equation (2):

(2) $$\gamma_{SA}=\gamma_{SA}+\gamma_{LA}cos\theta$$

where *γ*_SA_, *γ*_LA_, and *γ*_SL_ are the solid–air, liquid–air, and solid–liquid interfacial tension, respectively; *θ* is the contact angle between the liquid and the solid.

The model of the contact angle on the substrate is shown in Figure 8c. The relationship between *θ* and the sectional size can be calculated by Equation (3):

(3) $$\theta =arctan\frac{2d}{H}$$

where *H* is the height and *d* is the width of the section. The unit time volume of the injection in the experiment is generally in pico-liters, the surface area is negligible with respect to the surface area of the substrate, and the relationship between the liquid height and the diameter of the drop bottom can be calculated approximately according to the height method, as in Equation (3).

**Figure 9:**
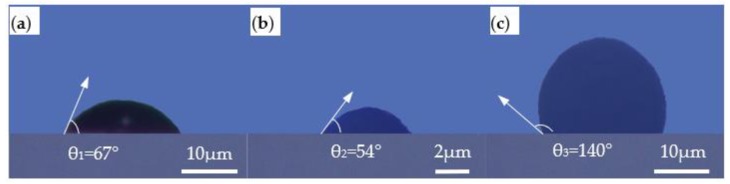
Contact angles of different materials at 20 °C: (**a**) MC-Wax; (**b**) PCL; (**c**) PMMA.

The contact angle *θ* generally relates to the surface energy of the three-phase medium according to Equation (2), and the surface energy is significantly influenced by the temperature. The morphology of the deposited materials at 20 °C are shown in Figure 9. Under the same printing parameters, the contact angle of PMMA is apparently larger than MC-Wax and PCL, meaning it can be used to fabricate large-area molds with high aspect ratios, while PCL produces higher resolution printing.

**Figure 10:**
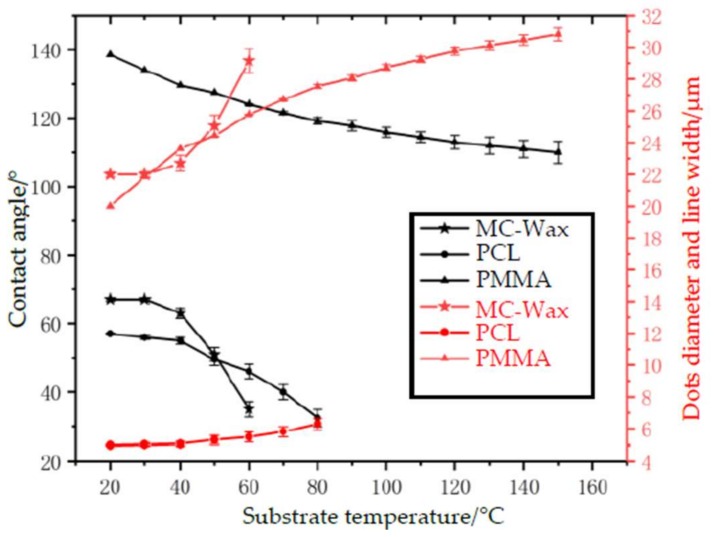
Effect of substrate temperature on printing characteristics.

In order to obtain a better surface morphology and realize multilayer accumulation in the experiment, auxiliary substrate heating was required. As the substrate heats up, the surface energy between the material and the substrate decreases, and the contact angle of the fused polymers with the surface of the substrate and the line width also decreases, as shown in Figure 10. As the temperature rises, the contact angle between the fused polymers and the substrate shrinks, and the ratio of the height to the dot diameter and line width decreases, resulting in decreased height and increased dot diameter and line width.

## 4. Research Application

Based on the differences in heating temperature, viscosity, wettability, and tensile properties, the three materials above can be applied to different fields. For example, for the low-viscosity material MC-Wax, through adjusting the pulse time to control the droplet injection, a microdot structure can be fabricated. When combined with drop-on-demand printing and layered superposition technology, EFD μ-3D printing can be used to produce three-dimensional solids with complex morphology. PCL has good biodegradability, excellent tensile properties, and good adhesion to the substrate. With appropriate electric field strength and printing speed, the minimum line width reached below 5 μm, which has obvious advantages in the manufacturing of high-resolution mesh structures, such as biological stents. As for PMMA, which maintains good wettability at high temperatures, when the glass substrate was heated to 100 °C, the contact angle was still up to 120°, and the aspect ratio reached 0.8, meaning it can be applied to fabricate large-area aspect ratio molds.

Through theoretical analysis, numerical simulation, and experimental research, the feasibility of electric-field-driven EFD μ-3D printing technology was verified. The optimal printing parameters for different viscosity materials were determined, as shown in Table 2. Research applications for different materials were carried out by using the optimal parameters in Table 2.

### 4.1. Microarray and Microstructure by drop-on-demand (DOD) printing

**Figure 11:**
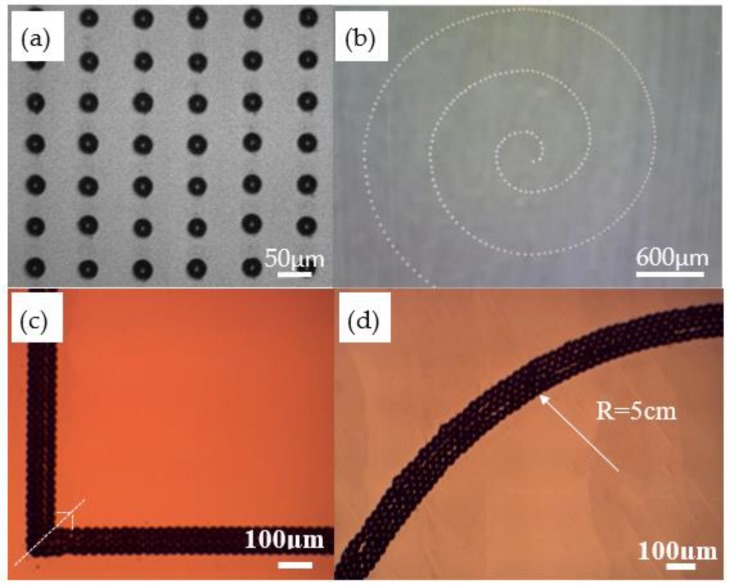
Printed MC-Wax microarray: (**a**) printed microarray with dot pitch measuring 80 μm; (**b**) printed helix; (**c**) printed high fill rate corner construction; (**d**) printed high fill rate fillet construction.

**Figure 12:**
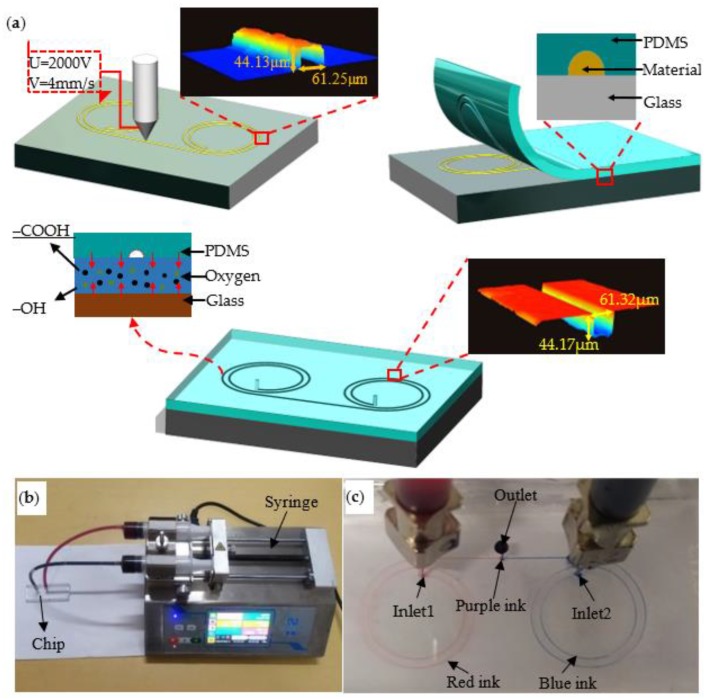
Fabrication of microfluidics chip: (**a**) schematic of microfluidic chip fabrication, PDMS = Polydimethylsiloxane; (**b**) microfluidics chip injection system ,including LEAD FLUID TYD01-02 dual-channel syringe pump (Nanjing, China) and the prepared microfluidics chip; (**c**) mixing and rendering of microfluidics chip.

MC-Wax is widely used in additive manufacturing as a styling and support material. It is also widely used in investment casting due to its low melting point and easy removal. The current MC-Wax printing method consists of fused deposition modeling and piezo-driven printing techniques, which have higher limits on the nozzle diameter and lower printing resolution [36]. In the experiments based on EFD μ-3D printing technology, the minimum dot diameter was reduced to 20 μm by using a 200 μm inner diameter metal nozzle. DOD printing can be achieved by coordinating the pulse frequency, duty cycle, and discharge position to fabricate microscale complex structural molds, and the printed MC-Wax microarrays are shown in Figure 11. Combined with layered manufacturing theory, a ten-micron microfluidic chip sample was prepared and the preliminary verification proved that it has the basic features of a microfluidic chip, as shown in Figure 12.

### 4.2. Microscale Mesh Structures

**Figure 13:**
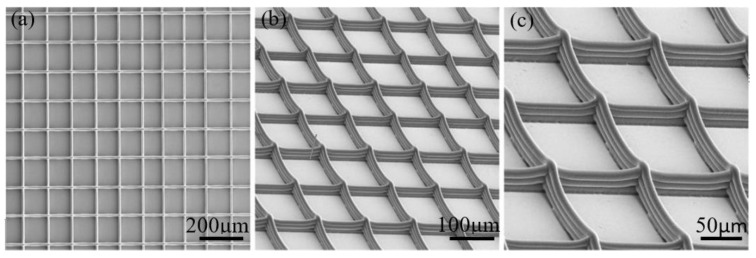
Printed biological tissue scaffold (4 mm × 4 mm): (**a**) overall picture; (**b**) a partially enlarged scanning electron miscroscopy (SEM) image of (a); (**c**) a parially enlarged SEM image of (b).

Three-dimensional microscale mesh structures have a wide range of engineering applications in the field of bioengineering. Engineering scaffolds are three-dimensional porous structures that transport nutrients and excretion metabolites for cell growth. Ideal engineering scaffolds must have controlled porosity, pore size, and pore distribution, which determine the supply of oxygen and nutrients to the cells in the scaffold. EFD μ-3D printing technology is used to control the high-resolution filament distribution of the jet. At the same time, the temperature of the tip and the substrate temperature are adjusted to achieve control of the filament solidification speed to control the sag of the filament, thereby achieving high-quality, microscale grid, three-dimensional structure controllable printing. Printed mesh structures can be used in scaffold fabrication for tissue engineering, as shown in Figure 13.

### 4.3. Large-Area Microscale Master Mold

**Figure 14:**
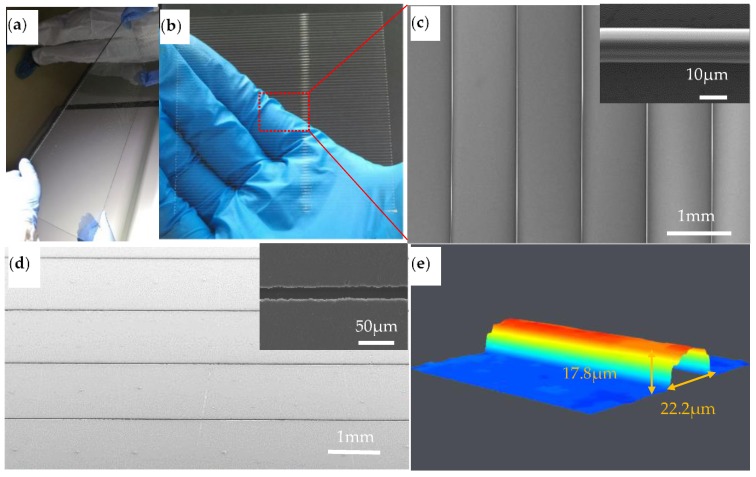
PMMA convex micromold printed using electric field-driven μ-3D printing: (**a**) printed PMMA large-area convex micromold (1200 mm × 800 mm); (**b**) printed PMMA large-area convex micromold (100 mm × 100 mm); (**c**) a partially enlarged SEM image of (b); (**d**) a replicated PDMS mold; (**e**) a partially enlarged volume microscope image of (**c**).

The manufacture of a large-area microscale mold is a challenging problem. PMMA has excellent optical properties, corrosion resistance, small shrinkage, and aging resistance, and can be used to prepare high aspect ratio and high-precision molds. In recent research, large-area micro- and nanoscale PMMA convex molds have been used to fabricate high-performance silver mesh for transparent glass heaters with UV-assisted microtransfer [37]. The PMMA convex micromold was printed by electric field-driven μ-3D printing with a minimum line width of 20 μm. As shown in Figure 14, the aspect ratio is 0.8 (ratio of height to width), enabling the fabrication of large-area microstructures at low cost and with high efficiency.

## 5. Conclusions

In this work, a new high-efficiency manufacturing technique for molds is proposed based on EFD μ-3D printing of fused polymers. Through theoretical analysis of the forming principle, numerical simulation, experimental research, and systematic study of the actual application, the following conclusions are obtained:

(1) The EFD μ-3D printing generates a self-excited electrostatic field to achieve the microscale 3D printing of fused polymers. It breaks through the requirements and limitations of conventional electrohydrodynamic jet printing for height formation, material type, substrate conductivity, and flatness, especially for large size, conformal, and curved 3D printing, further enhancing the stability of the printing process, enabling low-cost, high-resolution 3D printing for fused polymers.

(2) EFD μ-3D printing technology is used to fabricate fused polymers into convex mold. Based on the differences in heating temperature, viscosity, wettability, and tensile properties of the three materials, they can be applied to different fields.

Microarrays and three-dimensional solids with complex morphologies made from low viscosity materials, such as MC-Wax, are fabricated by drop-on-demand printing and layered superposition principle. PCL materials have good tensile properties and the minimum printing line width is 5 μm, which has obvious advantages in the production of high-resolution mesh structures, such as biological stents. PMMA can maintain good substrate wettability under high temperature.

When the substrate is glass and the heating temperature is 100 °C, the contact angle can still reach 120°, and the aspect ratio can reach 0.8, which is convenient in the manufacturing of large aspect ratio convex molds. The printing material does not require pretreatment, which is advantageous as it reduces the manufacturing cost of the mold and simplifies the manufacturing process.

(3) During the printing process, as the printing speed increases, the line width decreases accordingly, and the maximum speed is determined by the tensile properties of the fused material. The printable electric field strength is in the range of 7.6–11.6 × 10^6^ N/m, achieving continuous and stable printing. As the electric field strength increases, the dot diameter and line width first increase and then decrease, with slight variation due to the difference in viscosity. The larger the viscous force, the greater the magnitude of the change. The dot diameter and line width are affected by the substrate heating temperature. The higher the temperature, the smaller the contact angle between the liquid and the substrate, and the liquid spreads further on the substrate surface, resulting in an increase in line width and a decrease in aspect ratio.

## Figures and Tables

**Table 1:** Polymer parameters for EFD μ-3D printing.

Property	MC-Wax	PCL	PMMA
Melting Point (°C)	80	100	200
Density (g/cm³)	0.82	1.146	1.18
Relative Permittivity	2.0	2.5	2.87
Relative Molecular Mass	500–1000	56,000	80,000–120,000
Viscosity (mPa·s)	17.2 (80 °C)	17,500 (100 °C)	36,000 (200 °C)
Chemical Formula	C_30_H_50_O_2_	(C_6_H_10_O_2_)_n_	(CH_2_C(CH_3_)(COOCH_3_))_n_

**Table 2:** Printing parameters of different materials.

Material	Voltage	Temperature	Frequency	Distance	Print Speed
MC-Wax	2000 V	75 °C/80 °C	50 Hz, 33%	500 μm	4 mm/s
PCL	1200 V	95 °C/100 °C	—	200 μm	20 mm/s
PMMA	1300 V	220 °C/230 °C	—	200 μm	20 mm/s
